# Fibrinogen Gamma Chain Promotes Aggregation of Vesicular Stomatitis Virus in Saliva

**DOI:** 10.3390/v12030282

**Published:** 2020-03-04

**Authors:** Valesca Anschau, Rafael Sanjuán

**Affiliations:** Institute for Integrative Systems Biology (I2SysBio), Universitat de València-CSIC, Paterna, 46980 València, Spain; valesca.anschau@uv.es

**Keywords:** vesicular stomatitis virus, collective infectious units, viral transmission, virion aggregation, fibrinogen, label-free proteomics

## Abstract

The spread of viruses among cells and hosts often involves multi-virion structures. For instance, virions can form aggregates that allow for the co-delivery of multiple genome copies to the same cell from a single infectious unit. Previously, we showed that vesicular stomatitis virus (VSV), an enveloped, negative-strand RNA virus, undergoes strong aggregation in the presence of saliva from certain individuals. However, the molecular components responsible for such aggregation remain unknown. Here we show that saliva-driven aggregation is protein dependent, and we use comparative proteomics to analyze the protein content of strongly versus poorly aggregating saliva. Quantitative analysis of over 300 proteins led to the identification of 18 upregulated proteins in strongly aggregating saliva. One of these proteins, the fibrinogen gamma chain, was verified experimentally as a factor promoting VSV aggregation in a dose-dependent manner. This study hence identifies a protein responsible for saliva-driven VSV aggregation. Yet, the possible involvement of additional proteins or factors cannot be discarded.

## 1. Introduction

In recent years, evidence has accumulated that viruses can disseminate collectively via different types of multi-virion structures [1,2]. For instance, hepatitis A virus [3], enteroviruses [4], marseilleviruses [5], norovirus [6], and rotaviruses [7] can disperse at the intercellular and interhost levels as pools of viral particles cloaked in lipid microvesicles. Baculoviruses [8] and cypoviruses [9] disperse inside occlusion bodies constituted by a protein matrix of polyhedrin/granulin harboring multiple virions. In addition, viruses can be induced to aggregate by changes in pH, salt concentration, and concentration of cations [10]. For instance, aggregates of MS2 coliphage can be induced in a pH-dependent manner [11].

We have previously shown that vesicular stomatitis virus (VSV) forms abundant virion aggregates in the presence of saliva from certain donors [12]. Saliva is a natural shedding route for VSV [13,14,15], suggesting that virion aggregation could occur during interhost transmission. Other rhabdoviruses such as rabies viruses are also transmitted through saliva and share essential structural properties with VSV, suggesting that saliva-driven aggregation could also be involved in the interhost transmission of these viruses. Despite the known antiviral properties of saliva, a large number of viruses retain infectivity in saliva [16,17,18,19,20]. In fact, we previously showed that saliva-driven aggregation has a positive effect on early virus production, suggesting a fitness advantage of aggregation [21]. Therefore, unraveling saliva–virus interactions may have important implications for understanding virus biology and transmissibility. 

Although saliva has a potent aggregating effect on VSV and despite aggregation having implications for viral fitness, the components responsible for saliva-driven aggregation remain uncharacterized. Moreover, our previous work [12] showed that saliva obtained from different donors has widely different effects on VSV aggregation, suggesting that one or several saliva components present at variable concentrations among individuals are responsible for VSV aggregation. Here, we show that the saliva components driving virion aggregation have a proteinaceous nature. Then, using proteomic analysis, we identify saliva proteins differentially expressed in individuals with different VSV aggregating capability, and we experimentally show that one of these proteins, fibrinogen gamma-chain (FGG), actually promotes VSV aggregation.

## 2. Materials and Methods 

### 2.1. Virus and Cells 

VSV-mCherry and VSV-eGFP were obtained from an infectious cDNA clone in our laboratory. BHK-21 fibroblasts were obtained from the American Type Culture Collection and cultured from low-passage stocks in Dulbeco’s modified Eagle’s medium (DMEM) supplemented with 10% fetal bovine serum (FBS) at 37 °C in a 5% CO_2_ humidified incubator, and tested mycoplasma negative by PCR.

### 2.2. Virion Purification

Stocks of VSV-mCherry, VSV-eGFP, and VSV-A3853C were prepared by inoculating eight BHK-21 confluent T175 flasks at virus/cell ratio of of 0.01 plaque forming units (PFU) per cell. Supernatants were collected upon appearance of the first obvious cytopathic effects (∼20 h.p.i.). Cellular debris was removed by spinning (780 g, 5 min) and supernatants were filtered (0.22 µm). Virions were then pelleted at 30,000 g, 4 °C, 90 min. The pellet was resuspended in 2 mL NaCl 0.6%, Tris 0.6%, EDTA.Na2.2H2O 0.02% buffer at pH 7.4, laid on a 5%–50% iodixanol (Optiprep, Sigma-Aldrich, Misuri, USA) gradient in thin-walled tubes and ultracentrifuged at 160,000 g, 4 °C, for 90 min. Approximately 700 µl of a whitish band generated at mid-gradient was collected, aliquoted, and stored immediately at –70 °C. 

### 2.3. Virus Titration

Confluent BHK-21 monolayers were inoculated with various dilutions of the viruses, incubated for 45 min (37 °C; 5% CO_2_) and overlaid with DMEM, supplemented with 2% FBS containing 0.5%–0.6% agar. After 20−22h, cells were fixed with 10% formaldehyde, stained with 2% crystal violet, and plaques were counted.

### 2.4. Saliva Collection

Participants were advised not to eat, drink, or brush teeth 1 h before sampling. All saliva samples were collected between 9 a.m. and 11 p.m. to minimize day/night variability. Thirteen samples were taken from healthy individuals of either sex aged between 20 and 40. Unstimulated whole saliva was collected from all participants. Saliva samples were kept on ice and thereafter were centrifuged at 14.000 g for 20 min at 4 °C to remove insoluble materials, cell debris, and food remnants. The supernatant of each sample was collected and filtered through a 0.45 μm cellulose filter to remove bacteria, aliquoted, and stored at –70°C until use. All donors gave informed consent following the ethical guidelines of the Universitat de València. To test for saliva-driven aggregation, gradient-purified virions (10^9^ PFU/mL) were mixed 1:10 with saliva, incubated at 37 °C for 1 h, and diluted conveniently to infect cells (ca. 1000-fold).

### 2.5. Plasma and Serum Collection

Blood samples were collected in EDTA-coated tubes and human plasma was prepared by centrifugation (2000 g, 10 min, 4 °C). Serum was collected without EDTA-coated tubes and was prepared by the same centrifugation protocol. Plasma and serum were stored at –70 °C until analysis. The procedure for collecting plasma and serum was performed by the Centro de Transfusiones de la Comunidad Valenciana, Valencia, Spain. All donors gave informed consent following the ethical guidelines of the Universitat de València.

### 2.6. Automated Fluorescence Microscopy

Confluent BHK-21 cells in 6-well and 12-well dishes were inoculated with VSV and kept in an IncuCyte S3 Live-Cell Analysis System (Essen BioScience, Michigan, USA) at 37 °C and 5% CO_2_. Images were acquired using phase contrast, green, and red channels at 4× magnification and foci were counted manually.

### 2.7. Flow Cytometry Co-Fluorescence Assay

Cells were inoculated at ca. 0.1 PFU/cell and after 45 min at 37 °C and 5% CO2, cultures were overlaid with DMEM 1× supplemented with 2% FBS and incubated for 6 h (first infection cycle). Cells were then detached using trypsin-EDTA, resuspended in DMEM 1× containing 10% FBS, washed with cold PBS by centrifugation (700 g, 5 min, twice), resuspended in 1 mL 4% paraformaldehyde (PFA) for fixation and incubated overnight at 4 °C. PFA was removed and washed with cold PBS by centrifugation (700 g, 5 min, twice), cells were resuspended in 500 µL PBS and about 100,000 events per sample were analyzed in a Becton Dickinson LSRFortessa flow cytometer equipped with 488 and 561 nm lasers for eGFP and mCherry, respectively. Controls of non-infected cultures as well as of cultures singly infected with VSV-mCherry or VSV-eGFP were run in parallel and used to delineate quadrants manually. 

### 2.8. Probabilistic Model for Inferring the Fraction of Infectious Particles in Aggregates 

Flow cytometry cell counts were used to infer the fraction of virions bound to other virions in the inoculum, as described previously [12]. Briefly, we considered two types of single particles: red and green, with frequencies R and G, respectively. In addition, we considered three possible types of viral aggregates: red/red ($C_{\mathrm{rr}}$), green/green ($C_{\mathrm{gg}}$), red/green ($C_{\mathrm{rg}}$). The total frequency of aggregates was thus $C=C_{\mathrm{rr}}+{\;C}_{\mathrm{gg}}+C_{\mathrm{rg}}$, and since higher-order aggregates were not considered, R+G+C=1. Flow cytometry provided the observed proportions of non-infected ($P_{0}$), mCherry-only positive ($P_{R}$), GFP-only positive ($P_{G}$), and mCherry/GFP doubly fluorescent ($P_{\mathrm{RG}}$) cells. We inferred the virus/cell ratio at inoculation as $\exp\left(-P_{0}\right)$. Then, the fraction of cells receiving one ($P_{1}$) or two $(P_{2}$) infectious units was estimated using a Poisson distribution with mean equal to this inferred virus/cell ratio. The observed values $P_{R}$, $P_{G}$, and $P_{\mathrm{RG}}$ depended on model parameters as follows: $P_{R}=P_{1}\left(R+C_{\mathrm{rr}}\right)+{\;P}_{2}\left(R^{2}+2{\mathrm{RC}}_{\mathrm{rr}}+C_{\mathrm{rr}}^{2}\right)$, $P_{G}=P_{1}\left(G+C_{\mathrm{gg}}\right)+{\;P}_{2}\left(G^{2}+2{\mathrm{GC}}_{\mathrm{gg}}+C_{\mathrm{gg}}^{2}\right)$, and $P_{\mathrm{RG}}=P_{1}C_{\mathrm{rg}}+{\;P}_{2}\left(1-{\;R}^{2}-2{\mathrm{RC}}_{\mathrm{rr}}-C_{\mathrm{rr}}^{2}-{\;G}^{2}-2{\mathrm{GC}}_{\mathrm{gg}}-C_{\mathrm{gg}}^{2}\right)$. Furthermore, in a well-mixed virion suspension, the frequencies of each type of aggregate should obey the following proportions: $C_{\mathrm{rr}}/C={\;R}^{2}/{\left(R+G\right)}^{2}$, $C_{\mathrm{gg}}/C={\;G}^{2}/{\left(R+G\right)}^{2}$, and $C_{\mathrm{rg}}/C=2\mathrm{RG}/{\left(R+G\right)}^{2}$. Hence, parameters C, $C_{\mathrm{rr}}$, $C_{\mathrm{gg}}$, and $C_{\mathrm{rg}}$ could all be expressed in terms of R and G such that, in the model, $P_{R}$, $P_{G}$, and $P_{\mathrm{RG}}$ depended solely on R and G. A numerical solution was obtained by finding the values of R and G that minimized the sum of squared residuals between predicted and observed cell counts, and the proportion of aggregates was then obtained as C=1−R−G. The estimated proportion of infectious particles in aggregates, 2C/ (2C+ R+G), is reported in the text.

### 2.9. Reduction, Alkylation, and Digestion of Saliva Samples

Saliva from each experimental group was processed independently and in triplicate. Prior to digestion, saliva protein concentration was determined by the BCA assay Thermo Fisher Scientific, Massachusetts, USA) using a small sample aliquot. After dilution of samples with 50 mM ammonium bicarbonate buffer, cysteine residues were reduced with 2 mM dithiothreitol (DTT) at 60 °C for 20 min, and alkylated with 5.5 mM iodoacetamide (IAA) in the dark and at room temperature for 30 min. IAA excess was then neutralized by adding 10 mM DTT and incubated for 30 min at room temperature. Next, proteins were digested with 250 ng of sequencing-grade modified trypsin ((Promega, CA, USA) overnight at 37 °C. After acidification using 1% trifluoroacetic acid (TFA), the resulting peptides were concentrated in a vacuum concentrator to reach 300 ng/µL.

### 2.10. LC-MS/MS

Approximately 2.5 µg of each sample were loaded onto a trap column (NanoLC Column, 3 µm C18-CL, 350 µmx 0.5 mm; Eksigent, CA, USA) and desalted with 0.1% TFA at 3 µL/min during 5 min, and then onto an analytical column (LC Column, 3 µm C18-CL, Nikkyo, Tokyo, Japan) equilibrated in 5% acetonitrile (ACN), 0.1% FA (formic acid). Peptides were eluted with a 5%–35% linear gradient of solvent B in solvent A for 60 min (A: 0.1% FA; B: ACN, 0.1% FA) at a flow rate of 300 nL/min. Purified peptides were analyzed in a nanoESI qQTOF (5600 TripleTOF, Applied Biosystems Sciex, CA, USA), ionized by applying 2.8 kV to the spray emitter. Raw data files were analyzed with MaxQuant 1.6.3.3. MS/MS spectra were searched against the human and VSV complete proteome sequences obtained from Uniprot and a set of commonly observed contaminants. Precursor mass tolerance was set to 0.07 Da and 0.006 Da for the first search. The maximum precursor ion charge state used for searching was 7. Carbamidomethylation of cysteines was searched as a fixed modification, while oxidation of methionines and acetylation of protein N-terminal were searched as variable modifications. Enzyme was set to trypsin in a specific mode and a maximum of two missed cleavages was allowed for searching. The target-decoy-based false discovery rate (FDR) filter for spectrum and protein identification was set to 1%. The retention times of all analyzed samples were linearized with the “Match between runs” feature of MaxQuant [22]. Data were further analyzed with Perseus 1.5.2.12 [23]. First, calculated peptide intensities were log_2_-transformed and normalized across samples to account for systematic errors. Proteins from the reverse database, proteins only identified by site, and contaminants were removed. All statistical analyses were performed using t-tests with Benjamin–Hochberg correction cut off set at FDR 0.05. A *p*-value <0.05 was considered to indicate significant differences.

### 2.11. ELISA

The ELISA was performed using the Human Fibrinogen SimpleStep ELISA kit (Abcam, Cambridge, UK) according to the manufacturers’ instructions.

## 3. Results

### 3.1. VSV Aggregation is Promoted by Human Saliva in a Variable and Protein-Dependent Manner

**Figure 1:**
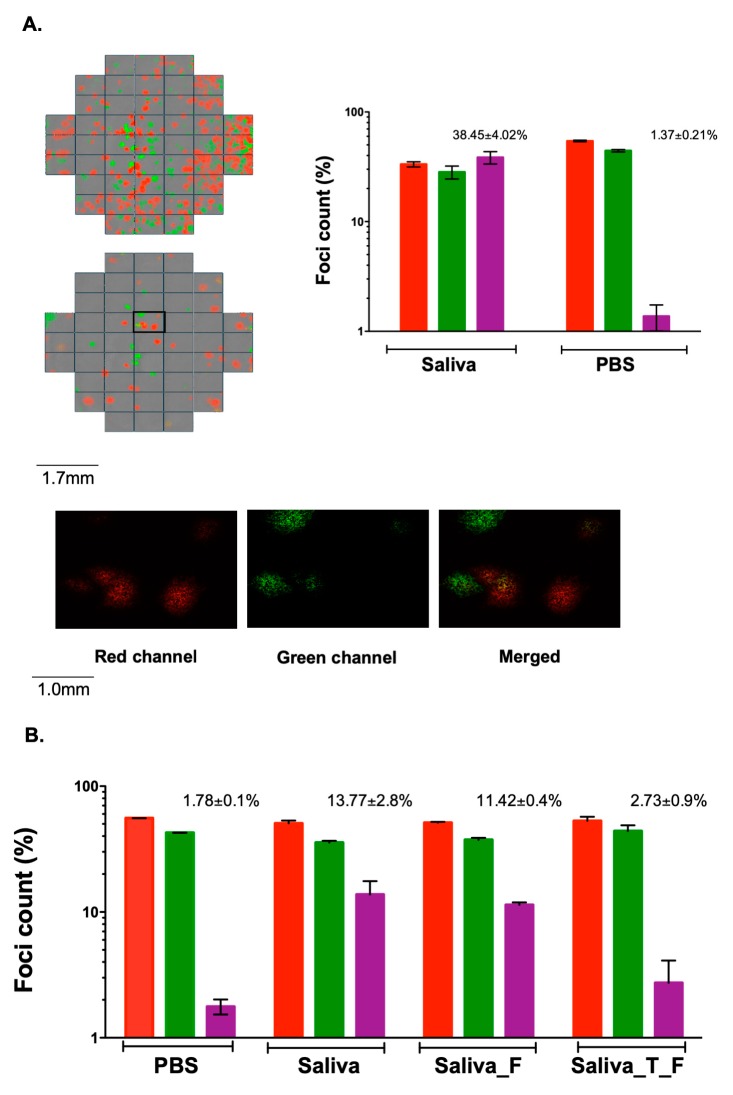
Effect of saliva on vesicular stomatitis virus (VSV) aggregation as determined by foci analysis. (**A**) Fluorescence microscopy images of BHK-21 cells inoculated with VSV-mCherry and VSV-eGFP. A 1:1 mix of these two viruses was incubated for 1h at 37 °C with PBS or human saliva from donor P6, used for inoculating cells, and images were taken at 13 hpi. Bars indicate the percentage of foci positive for eGFP only (green), mCherry only (red), and doubly fluorescent foci (purple). Error bars represent the SEM from three technical replicates. (**B**) Effect of trypsin on saliva-driven VSV aggregation. Prior to inoculation, the 1:1 mix of VSV-mCherry and VSV-eGFP was incubated for 1 h at 37 °C in PBS, in saliva from donor P8, in P8 saliva previously mixed with FBS (Saliva_F), or in P8 saliva treated with trypsin (2 h at 37 °C) followed by trypsin inactivation with FBS (Saliva_T_F). Error bars represent the SEM from three technical replicates.

In previous work, we used various methods including differential centrifugation, dynamic light scattering, nanoparticle tracking analysis, and transmission electron microscopy to characterize the virion aggregation process in VSV [12]. In particular, we established a co-fluorescence-based assay to show that aggregates can deliver multiple viral genome copies to cells, and to quantify aggregation. This assay uses two VSV recombinants encoding mCherry or eGFP for inoculating cells at a low ratio of PFU to cells. Under these conditions, coinfection with two or more viral genomes is infrequent except if virions are aggregated. Hence, virion aggregation can be quantified by assessing the percentage of cells or infection foci that are positive for both fluorescent markers. Here, we used this approach to test saliva samples collected from thirteen volunteers. Purified VSV-mCherry and VSV-eGFP virions were prepared separately and co-incubated in human saliva (10^8^ PFU/mL each) for 1 h at 37 °C. This mix was used to inoculate baby hamster kidney (BHK-21) cell monolayers. As a control, we performed identical assays using PBS instead of saliva. At 13 h post inoculation (hpi), we evaluated the appearance of doubly fluorescent infection foci as a readout for virion aggregation, using automated fluorescence microscopy. As expected, doubly fluorescent foci showed low frequencies in cultures inoculated with the untreated virus (0.69% ± 0.12% and 3.18% ± 0.24% in two independent experiments). In the presence of saliva from certain donors but not others, a larger fraction of the foci produced were positive for both eGFP and mCherry, the percentage of doubly fluorescent foci ranging from 1.25% ± 0.37% to 38.5% ± 4.0% (Table 1, Figure 1A). This confirmed that saliva can promote VSV aggregation and this ability is highly variable among donors.

Since the VSV surface glycoprotein G can trigger membrane–membrane interactions at low pH [11], we first measured salivary pH in order to test whether this might explain among-donor variability in saliva-induced VSV aggregation. However, we did not find any significant association between salivary pH and the formation of aggregates (Table 1). Indeed, VSV aggregation occurred efficiently at physiological pH values of 7.4.

We then set out to explore whether aggregation was protein dependent. For this, we treated human saliva with trypsin for 2 h at 37 °C and quenched the reaction with FBS. We then incubated virions with trypsin-digested saliva. Under these conditions, foci analysis showed minimal aggregation (1.78% ± 0.1% and 2.73% ± 0.9% in two independent experiments) when compared with a positive control using non-digested saliva (13.77% ± 2.8%; Figure 1B). This suggests that proteins present in saliva are required for VSV aggregation.

### 3.2. Identification of Differentially Expressed Saliva Proteins Associated to VSV Aggregation

Based on the above results, we set out to determine the expression levels of proteins present in poorly aggregating versus strongly aggregating saliva from different donors. Using our foci analysis data (Table 1), donors P7, P11, and P13 were selected as poor aggregators (Group 1) whereas P6, P8, and P10 (Group 2) were selected as strong aggregators. In addition, saliva from Group 2 was used for aggregating VSV and then centrifuged at low speed (10 min, 5000 g) to pellet virion aggregates. Foci analysis showed that slow-speed centrifugation pelleted approximately half of the aggregates (Figure S1). In contrast, as shown previously, non-aggregated VSV particles do not pellet at this centrifugation speed [12]. The VSV-containing, centrifuged saliva from Group 2 donors constituted our Group 3. Our rationale for identifying saliva proteins responsible for VSV aggregation was that (i) they should be significantly enriched in Group 2 compared to Group 1 and (ii) they should not be significantly depleted in Group 3 compared to Group 2.

**Figure 2:**
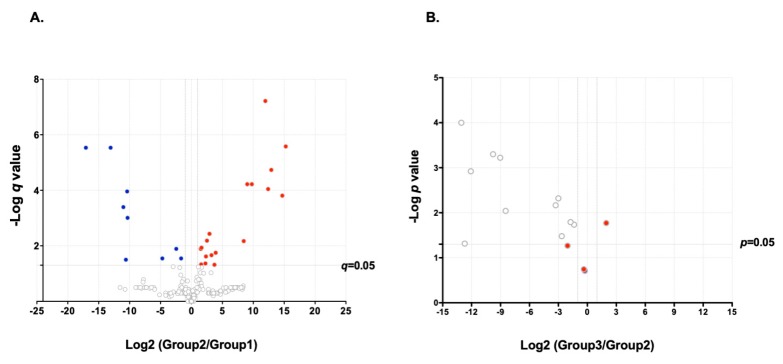
Volcano plots showing differential proteins expression. (**A**) Group 1 versus Group 2. Red dots: proteins upregulated in Group 2. Blue dots: proteins downregulated in Group 2. Selection criteria were (i) that abundance should differ by at least twofold in Groups 2 and 1 and that (ii) differences in abundance should be significant at the 0.05 level in a t-test adjusted for a 5% FDR after multiple testing (*q*-value < 0.05). (**B**) Group 3 versus Group 2. Red dots: proteins showing statistically indistinguishable or greater expression level in Group 3 compared to Group 2. Only proteins that were measurable in all 6 samples were used for this comparison. A List of all proteins is available from Table S2.

The nine samples (three for each Group 1, 2, and 3) were subjected to LC/MSMS proteomic analysis. This allowed us to identify 3303 unique peptides corresponding to 320 described proteins. From this list, we selected enriched or depleted proteins as those exhibiting a fold-change in abundance greater than 2.0 that was statistically significant (t-test: *p*-value adjusted for FDR, *q* < 0.05). We found nine depleted proteins in Group 2 compared to Group 1, while 18 were enriched (Figure 2A; Table S1). Focusing on the 18 proteins that were enriched in Group 2 relative to Group 1, we tested for differences between Groups 2 and 3, using a paired t-test (Figure 2B). For this, we used only proteins that were measurable in all 6 samples. Among the 18 candidate proteins, four showed no significant depletion in Group 3 compared to Group 2: fibrinogen gamma chain (FGG), Ig heavy chain variable region, rheumatoid factor D5 light chain, and transferrin.

Hence, these four proteins met our selection criteria for being potentially involved in VSV aggregation. We selected FGG and transferrin for further tests, but we did not analyze the Ig heavy chain variable region and rheumatoid factor D5 light chain further because these are antibodies showing high variability among individuals, and their analysis would thus require expression of each particular variant. We cannot at present confirm or discard their involvement in VSV aggregation.

### 3.3. Experimental Validation of VSV Aggregation

We used commercially available recombinant FGG and transferrin to test experimentally their ability to induce VSV aggregation. For this, 125 ng/mL of recombinant FGG or transferrin (final concentration) was added to saliva from donor P7, which per se exhibited very low aggregation capacity (1.25% ± 0.37% doubly fluorescent cells) and tested for changes in the formation of virion aggregates, as determined by the appearance of doubly fluorescent foci. We found no doubly fluorescent foci out of 108 analyzed for transferrin, suggesting no strong involvement of this protein in VSV aggregation, although the number of foci analyzed was relatively small. For FGG, we found six doubly fluorescent foci out of 286 analyzed (2.1%). In addition, Gene Ontology terms indicate that FGG can be involved in protein-containing complex assembly (GO: 0065003), which may be relevant for VSV aggregation. In contrast, transferrin is associated mainly with iron transport (GO:0006826) and cellular iron homeostasis (GO: 0006879) and, consequently, is less likely to play a direct role in virus aggregation.

**Figure 3:**
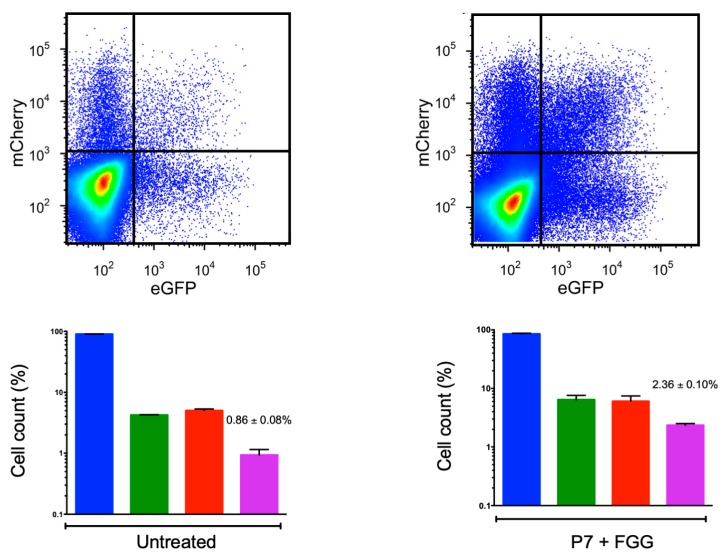
Fibrinogen gamma-chain (FGG) effect on vesicular stomatitis virus (VSV) aggregation assayed by flow cytometry. Top: Scatter plots (>100,000 events) showing non-infected (blue), mCherry-positive (red), eGFP-positive (green), and doubly fluorescent (purple) cells at 6 hpi in cultures inoculated with a 1:1 mix VSV-mCherry VSV-eGFP (0.1 PFU/cell). Prior to inoculation, the mix was either incubated (1 h, 37 °C) in saliva from donor P7 or in P7 saliva supplemented with recombinant FGG (125 ng/mL). Scatter plot data show one out of the three technical replicates conducted. Bottom: histograms showing the mean percentage of foci positive for eGFP only (green), mCherry only (red), and doubly fluorescent foci (purple) from three technical replicates (error bars: SEM).

We hence set out to test for FGG-driven aggregation more accurately. For this, we inoculated cells at ca. 0.1 PFU/cell ratio and used flow cytometry to count the numbers of non-infected, VSV-eGFP-positive, VSV-mCherry-positive, and doubly fluorescent cells within the time range of the first infection cycle (6 hpi), as described previously [12]. This assay is equivalent to the foci enumeration assay used above but allows counting many more infection events (>100,000 cells). P7 saliva supplemented with FGG showed a significantly higher number of doubly fluorescent cells (2.36% ± 0.10%) than non-supplemented P7 saliva (0.86% ± 0.08%; t-test: *p* < 0.001; Figure 3), albeit the effect of FGG on aggregation was modest.

Since co-fluorescent cell counts are highly sensitive to variations in the PFU/cell ratio (higher ratios allow for more coinfections with independent virions by chance), we analyzed these data using a previously described probabilistic model that allows estimating the actual fraction of aggregates independent of the PFU/cell ratio [12]. This model makes a prediction of the fraction of doubly fluorescent cells as a function of the PFU/cell ratio, the eGFP/mCherry virus ratio, and the fraction of infectious units constituted by aggregates (assuming two infectious particles per aggregate for simplicity, see Methods). The model parameters were adjusted to the observed fraction of doubly fluorescent cells by the least-squares method, providing an estimate of the fraction of infectious particles forming aggregates. In FGG-supplemented saliva, this fraction was estimated to be 24.9% ± 3.6%, compared to 11.9% ± 1.1% in non-supplemented P7 saliva (t-test: *p* = 0.025), again providing evidence that FGG promotes VSV aggregation.

**Figure 4:**
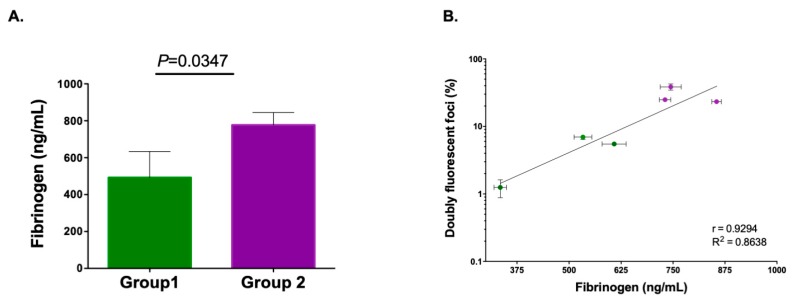
Relationship between viral aggregation and fibrinogen levels. (**A**) Green and purple bars represent ELISA-determined fibrinogen levels in saliva from Group 1 and Group 2 donors (error bar: SEM). (**B**) Correlation between fibrinogen concentration and aggregation, as determined by foci co-fluorescence analysis. The line shows the least-squares regression fit.

We then used ELISA to quantify fibrinogen concentration in the six saliva samples used for the proteomic analysis. We obtained significantly higher fibrinogen concentrations in Group 2 (777 ± 39 ng/mL) than in Group 1 (492 ± 82 ng/mL; t-test: *p* = 0.0347; Figure 4A). Notably, P7 (the most poorly aggregating saliva) exhibited the lowest fibrinogen concentration (335 ± 15 ng/mL). Furthermore, we observed a significantly positive correlation between fibrinogen levels and saliva aggregation capacity, as determined by the log percentage of doubly fluorescent foci (*n* = 6, Pearson correlation: *r* = 0.9294 and *p* = 0.0073; Figure 4B). Indeed, a linear regression of the log fraction of doubly fluorescent foci against fibrinogen concentration explained the data relatively well (slope: 0.0028 ± 0.0006; y-intercept: -0.767 ± 0.364; *r^2^* = 0.864). Our findings are thus consistent with a dose-dependent effect of fibrinogen on VSV aggregation in saliva. We also noticed that the measured fibrinogen concentrations were high compared to the 125 ng/mL supplement of commercial FGG used above. This could explain why our FGG supplementation experiment had a relatively modest effect on aggregation (Figure 3). Actually, according the above linear regression, the expected effect of this FGG supplementation was 2.2-fold, consistent with the observations.

Finally, to seek for evidence supporting the effect of fibrinogen on VSV aggregation in other human fluids, we obtained both plasma and serum from donors. Fibrinogen is abundant (150–400 mg/dL) in blood plasma but infrequent in serum, except in patients with coagulation disorders such as congenital dysfibrinogenemia [24,25,26]. We selected three blood donors and used the flow cytometry co-fluorescence assay to compare the formation of VSV aggregates in plasma and serum obtained from the same donor. We found that the fraction of infectious particles in aggregates was low (<2%) in VSV incubated in serum, whereas this fraction ranged from 6.1% to 18.6% in plasma from the same donors (t-test: *p* < 0.01 for each of the three donors; Table 2).

## 4. Discussion

We have shown that human saliva can promote VSV aggregation, but that this ability varies strongly among donors. In a previous study, we also reported such variability in cow saliva [12]. Our unpublished observations indicate that the VSV aggregation capacity of samples taken from the same donor at different time points shows less variability than samples from different donors, suggesting among-individual diversity. Yet, we found no obvious factors (age, diet, smoking, concomitant diseases) explaining such differences. Here, to better understand possible molecular factors responsible for VSV aggregation in saliva, we have undertaken a proteomic analysis of three strongly versus three poorly aggregating saliva samples. We obtained differences for several proteins, of which FGG was a good candidate based on our selection criteria. ELISA-based fibrinogen quantitation, experiments with purified FGG, and plasma versus serum comparisons supported a role for fibrinogen in VSV aggregation.

However, our results do not imply that fibrinogen is the sole factor responsible for VSV aggregation in saliva. Additional proteins including antibodies could further contribute to this process. In general, proteins with several domains capable of interacting with other proteins or small molecules might be good candidates. These criteria are indeed met by fibrinogen, a binder protein capable of interacting with a large number of other proteins [27]. Twelve amino acids at C-terminal of the FGG chain are responsible for binding to platelet αIIbβ3 integrin, forming a bridge between activated platelets during the aggregation phase of platelet plug formation [28,29,30]. In addition, several binding sites have been described for growth factors and cytokines involved in inflammation and wound healing [31]. Fibrinogen also participates in defense against infections [32,33], for instance, by interacting with M1 surface proteins in *S. pyogenes* [34].

The mechanism whereby fibrinogen promotes VSV aggregation remains to be determined, though. The VSV surface glycoprotein G binds proteins of the LDL receptor family, which are used for virion attachment to the cell surface and entry [35]. VSV-G can also interact with phosphatidylserine [36]. This lipid is not present in the external leaflet of the plasma membrane in healthy cells and, consequently, is not used for initial binding and entry, but phosphatidylserine is probably involved in VSV-endosome interactions after virion internalization, which lead to membrane fusion and release of the ribonucleocapsid to the cytosol. We have also suggested recently that interactions between VSV-G and phosphatidylserine mediate virion aggregation in the extracellular milieu [12]. However, this process only occurred appreciably in highly concentrated purified virion suspensions. In contrast, saliva-driven aggregation does not require such high density of virions, suggesting a different molecular process, in which fibrinogen appears to play a role. Contacts might be established directly between VSV-G and fibrinogen. However, VSV-G is not the only surface-exposed protein in virions. Previous work has identified large numbers of host-encoded proteins embedded in the VSV envelope [37], which could also potentially interact with fibrinogen. Among these proteins, the STRING database [38] suggests that integrin β3, amyloid-beta A4, and fibronectin could interact with FGG. Of these, fibronectin is the most abundantly present at the VSV surface [37]. Fibronectin binds fibrin (a proteolytic product of fibrinogen) strongly, but not fibrinogen [39]. Therefore, at present, there is no obvious virion surface protein candidate for establishing interactions with fibrinogen and trigger the formation of viral aggregates.

In summary, we have provided lines of evidence establishing FGG as a protein involved in VSV aggregation, but the specific mechanism at play and the virion proteins interacting with FGG remain to be elucidated. In many cases, viral aggregation is not a well-studied phenomenon, and factors such as protein hydrophobicity, shape, accessible surface area, and residue preference, as well as salt concentrations, virion concentrations, etc., could play a role. Future work may further characterize aggregation in VSV and other viruses from the molecular point of view. This should help us understand the phenomenon of virus aggregation in human saliva and other bodily fluids, to better test whether theses interactions are beneficial for viral fitness or antiviral, and to clarify the evolutionary significance of viral aggregation.

## Figures and Tables

**Table 1:** Percentage (mean ± SEM, *n* = 3) of doubly-fluorescent foci in cell monolayers inoculated with a 1:1 mixture of vesicular stomatitis virus (VSV)-mCherry and VSV-eGFP with or without (PBS) prior incubation in saliva.

Donor	Saliva Incubation	PBS Control	t-Test *p*-Value	Salivary pH	Gender	Age
**P1^1^**	6.91 ± 0.61	0.64 ± 0.08	< 0.001	6.4	F	32
**P2**	10.03 ± 0.73	0.26 ± 0.26	< 0.001	7.4	F	24
**P3**	11.82 ± 0.62	0.63 ± 0.32	< 0.001	7.3	M	25
**P4**	19.79 ± 1.92	2.87 ± 0.34	< 0.001	7.2	M	31
**P5^2^**	23.92 ± 0.74	0.95 ± 0.17	< 0.001	7.4	M	25
**P6**	38.46 ± 2.84	1.38 ± 0.21	< 0.001	7.0	F	27
**P7**	1.26 ± 0.38	0.34 ± 0.09	0.0764	6.4	M	22
**P8**	23.23 ± 1.23	1.65 ± 0.89	< 0.001	6.5	F	24
**P9**	20.85 ± 0.05	0.93 ± 0.14	< 0.001	7.2	F	34
**P10**	24.84 ± 1.76	1.23 ± 0.17	< 0.001	6.4	F	33
**P11**	6.96 ± 0.54	1.57 ± 0.25	< 0.001	7.0	M	40
**P12**	13.89 ± 1.02	0.79 ± 0.18	< 0.001	7.0	M	39
**P13**	5.37 ± 0.37	1.61 ± 0.06	< 0.001	6.0	F	37

^1^This donor was subsequently found to be diabetic and was excluded from further analyses. ^2^Saliva from this donor showed ample variability in aggregation capacity across days and was excluded.

**Table 2:** Percentage (mean ± SEM, *n* = 3) of infectious particles in aggregates for viruses incubated with serum or plasma from three different donors.

Donor	Serum	Plasma
**1**	2.46 ± 0.01	8.19 ± 0.01
**2**	1.85 ± 0.01	25.86 ± 0.02
**3**	1.79 ± 0.01	24.09 ± 0.02

## Supplementary Materials

The following are available online at https://www.mdpi.com/1999-4915/12/3/282/s1, Figure S1: Foci analysis of supernatant and pellet fractions following saliva-driven aggregation and low-speed centrifugation. Bars indicate the percentage of foci positive for eGFP only (green), mCherry only (red), and doubly fluorescent foci (purple). Error bars represent the SEM from three technical replicates. Table S1: Enriched and depleted proteins identified as those exhibiting a fold-change in abundance greater than 2.0 that was statistically significant (t-test: *p*-value adjusted for FDR, *q* < 0.05) in Group 2 compared to Group 1. Table S2: List of all proteins identified by LC/MSMS proteomic analysis (Excel format).
