# Supplementary material for: Fibrinogen Gamma Chain Promotes Aggregation of Vesicular Stomatitis Virus in Saliva
Source: Viruses. 2020 Mar 4;12(3):282. doi: 10.3390/v12030282 (PMC7150986; doi:10.3390/v12030282)
Supplement: Supplementary file 1 [file viruses-12-00282-s001.zip › viruses-715475.suppl zip/Supplementary data_v2.pdf]

Supplementary information

Table S1: Enriched and depleted proteins in Group 2 relative to Group 1.

Class	Entrez accession	Uniprot accession	Protein name
Enriched	FGA	A0A087WUA0	Fibrinogen alpha chain
	FGG	C9JEU5	Fibrinogen gamma chain
	IGKV2-40	A0A087X0Q4	Immunoglobulin kappa variable 2-40
	ALDH3A1	C9JMC5	Aldehyde dehydrogenase, dimeric NADP-preferring
	GPI	A0A2U3TZU2	Glucose-6-phosphate isomerase
	HP	J3QLC9	Haptoglobin
	A1BG	M0R009	Alpha-1B-glycoprotein
	N/A	A2MYD0	V1-17 protein
	PTGR1	Q5JVP2	Prostaglandin reductase 1
	APOA1	F8W696	Apolipoprotein A-I
	HSP90AA1	Q96HX7	HSP90AA1 protein
	V-kappa-3	A0N5G5	Rheumatoid factor D5 light chain
	IgH	A0A2U8J967	Ig heavy chain variable region
	SH3BGRL	D3DTE6	SH3 domain-binding glutamic acid-rich-like protein
	PZP	B2R950	pregnancy-zone protein (PZP)
	GRN	K7EKL3	Granulins
	YWHAZ	E5RGE1	14-3-3 protein zeta/delta
	TF	Q06AH7	Transferrin
Depleted	FCGBP	A0A286YF91	IgGFc-binding protein
	CPE	D6RF88	Carboxypeptidase E
	CES2	Q4G0E9	Carboxylic ester hydrolase
	IGLL5	A0A0B4J231	Immunoglobulin lambda-like polypeptide 5
	IGHG1	A0A0A0MS08	Immunoglobulin heavy constant gamma 1
	HEL-S-39	V9HWC6	Peptidyl-prolyl cis-trans isomerase
	HP	J3QR68	Haptoglobin
	CALML5	Q53H37	Calmodulin-like protein 5
	N/A	Q9HCC1	Single chain Fv

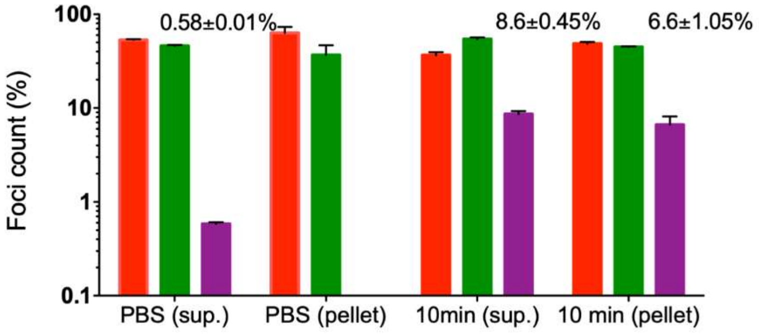

Figure S1. Virions were centrifuged after 1h incubation with saliva at 37 °C. The pellet and supernatant used for inoculating cells, and images were taken at 13 hpi. Bars indicate the percentage of foci positive for eGFP only (green), mCherry only (red), and doubly fluorescent foci (purple). Error bars represent the SEM from three technical replicates.
